# Factors Associated with Maternal Mortality from COVID-19 in Pernambuco, Brazil (2020–2021): A Case–Control Study

**DOI:** 10.3390/diseases14020071

**Published:** 2026-02-14

**Authors:** Tacilene Luzia da Silva, Cristine Vieira do Bonfim, Ulisses Ramos Montarroyos, Carlos Alexandre Antunes de Brito

**Affiliations:** 1 Postgraduate Program in Tropical Medicine, Center of Medical Sciences, Federal University of Pernambuco, Recife 50670-901, Brazil; 2 Directorate of Social Research, Joaquim Nabuco Foundation, Recife 52061-540, Brazil; 3 Institute of Biological Sciences, University of Pernambuco, Recife 50100-130, Brazil; 4 Department of Immunology, Autoimune Research Institute, Recife 52011-040, Brazil

**Keywords:** COVID-19, maternal mortality, SARS-CoV-2, case–control studies, pregnancy, postpartum period, pandemics, epidemiology

## Abstract

Background: The COVID-19 pandemic has contributed to the increase in maternal mortality due to the direct effects of the viral infection and the indirect effects caused by the overload of health services, and the resulting economic and social crises. This study aims to analyze sociodemographic, gestational, and clinical factors associated with maternal deaths from COVID-19 in Pernambuco between 2020 and 2021. Method: The study included 37 cases (deaths) and 112 controls (survivors). Crude and adjusted odds ratios were estimated using conditional and Firth’s penalized logistic regression models, respectively, to evaluate sociodemographic, gestational, and clinical factors. Results: In the bivariate analysis, the main factors associated with maternal death from COVID-19 were ≤8 years of schooling, the postpartum period, multiparity, oxygen saturation below 95%, obesity, and diabetes mellitus. The presence of fever and cough was associated with a lower probability of death. The independent factors that remained associated with maternal death were the postpartum period (*aOR*: 80.78; 95% CI: 16.54–394.37), parity ≥ 1 (*aOR*: 5.74; 95% CI: 1.16–28.22), and oxygen saturation below 95% (*aOR*: 7.16; 95% CI: 1.37–37.44), with fever acting as a possible protective factor (*aOR*: 0.08; 95% CI: 0.01–0.42). Factors such as obesity and diabetes were not independent predictors in the final multivariable model. Conclusions: The findings reinforce that maternal death is a multifactorial phenomenon. The relevance of this investigation lies in identifying clinical and obstetric vulnerability profiles in a region heavily impacted by the health crisis. Knowledge gained from past crises contributes to the improvement of public health strategies and clinical management protocols, aiming to mitigate preventable maternal deaths in future public health emergencies.

## 1. Introduction

Maternal mortality is a critical public health indicator, reflecting social inequalities and limited access to quality healthcare services [1]. The COVID-19 pandemic has contributed to a rise in global maternal mortality, driven by both direct clinical complications of SARS-CoV-2 infection and the indirect effects of healthcare system strain, exacerbated by socioeconomic crises [2]. Pregnant and postpartum women are particularly vulnerable to infection due to anatomical and physiological adaptations inherent to this period. These include altered immune function, diaphragmatic elevation, reduced respiratory capacity, and an increased risk of thrombotic events, all of which heighten susceptibility to severe forms of the disease, especially among women with comorbidities [3,4].

The pandemic exposed and deepened existing weaknesses in obstetric care, especially in low- and middle-income countries [5]. Disruptions to essential services and worsening social inequalities reversed progress in reducing maternal mortality [6]. In this context, maternal mortality rates in Latin America and Africa were higher than those in North America and Europe [2]. In Brazil, the Maternal Mortality Ratio (MMR) rose from 67 in 2020 to 107 per 100,000 live births in 2021 [7]. In the Northeast region, maternal mortality increased by 67% between 2020 and 2021 [8]. While in Pernambuco, the MMR increased from 48 to 73.7 deaths per 100,000 live births during the same period [9]. These differences were accentuated by disparities in access to control measures and healthcare resources, hindering an equitable response and delaying epidemiological control in more vulnerable areas [10].

Prior to widespread vaccine availability, non-pharmaceutical interventions served as the cornerstones of the pandemic response. Social distancing measures and restrictions on non-priority activities were pivotal to reducing the effective reproduction number (Rt) and flattening the epidemic curve, thereby preserving the operational capacity of healthcare systems. Subsequently, this scenario was transformed by the implementation of immunization; according to mathematical transmission models across 185 countries, vaccination programs in their first year were responsible for a global reduction of approximately 63% in expected deaths, averting an estimated 19.8 million lives lost [11]. This rapid decline in fatalities reflects both the direct protection of vaccinated individuals and the indirect effects of community transmission reduction, which are reinforced by non-pharmacological measures [12].

In Brazil, the initial lack of evidence associating COVID-19 with adverse maternal outcomes contributed to a delay in including pregnant and postpartum women as a priority group in the immunization plan [13,14,15]. However, the attenuation of maternal morbidity and mortality was achieved through the consolidation of the vaccination strategy for this group in mid-2021, with immunization being the decisive factor in altering the epidemiological profile [13,16].

Despite the global relevance of the topic, geographic disparities in scientific production and gaps in clinical-epidemiological details persist [2]. There is a paucity of data exploring the interaction of sociodemographic, gestational, and clinical factors that contributed to maternal death from COVID-19 in Pernambuco. Insufficient knowledge regarding these determinants limits the capacity to establish priorities, develop prevention policies, and respond to future public health emergencies.

Given this context, the present study seeks to investigate the sociodemographic, gestational, and clinical factors associated with maternal deaths from COVID-19 in Pernambuco between 2020 and 2021.

## 2. Materials and Methods

A matched case–control study was conducted using secondary data from pregnant and postpartum women with COVID-19. The study was carried out in the state of Pernambuco, located in Northeastern Brazil, which had an estimated population of 9.67 million in 2021, a population density of 89.6 inhabitants/km^2^, and a Human Development Index (HDI) of 0.719 [17]. These indicators contextualize the study population and their access to healthcare services, enabling comparisons with other regions sharing similar geographic and socioeconomic characteristics.

Eligible participants were pregnant or postpartum women residing in Pernambuco with COVID-19 confirmed by reverse transcription polymerase chain reaction (RT-PCR) recorded in the health information systems (HIS) between 2020 and 2021. The case group, in addition to meeting these criteria, consisted of women whose deaths were attributed to COVID-19 (underlying cause), as confirmed by a maternal mortality technical review committee.

Each maternal death in Brazil undergoes a detailed investigation by health surveillance services, covering prenatal care, delivery, and the postpartum period to identify causes and propose preventive measures. Findings are reviewed by technical committees and recorded in the standardized “Maternal Death Investigation Form–Summary, Conclusions, and Recommendations (summary-form)”, which consolidates information from multiple sources [18].

Cases in Pernambuco were identified and confirmed using state-level databases from two national systems: the Mortality Information System (SIM) and the Live Birth Information System (SINASC). The Maternal Death Investigation Form (summary-form; 42 records) served as the primary source for pregnancy-related data. Additionally, SIM provided the underlying cause of death and sociodemographic data (SIM; 46 records), using the Death Certificate (DC) as its data source. Meanwhile, SINASC was used to fill gaps in sociodemographic and obstetric variables by compiling data from Live Birth Certificates (LBC) [19].

Clinical data, including comorbidities, signs, and symptoms, were obtained from two information systems. The Influenza Epidemiological Surveillance System (SIVEP-Gripe) (627 records) was the main source, being a national platform for monitoring hospitalized cases of severe acute respiratory syndrome (SARS). To complement the variables unavailable in SIVEP-Gripe, the study also used Notifica-PE (53,954 records), a platform developed in Pernambuco during the pandemic designed for the notification of SARS cases and deaths. These systems record acute-onset symptoms. To ensure this distinction, the databases provide specific and independent fields for ‘Signs and Symptoms’ and ‘Pre-existing Comorbidities’. Laboratory confirmation of SARS-CoV-2 infection was obtained from the Laboratory Environment Manager (GAL) (26,480 records), a nationwide system for recording analyses relevant to health surveillance.

Data from the summary-forms were double-entered and validated to ensure database integrity. Duplicates, special characters, blank spaces, and inconsistent entries were removed. Utilizing Microsoft Excel 2010, sociodemographic, obstetric, and clinical information for each death was consolidated into a single, standardized database with no missing values for the selected variables.

To identify deaths across the different databases, a combination of identifiers—including full name, date of birth, date of death, mother’s name, and place of residence—was used. Records were considered confirmed matches when all variables matched exactly. Discordant pairs were manually reviewed and classified as true or false; only true pairs were retained for analysis.

The control group, in addition to meeting the previously mentioned criteria for residence and diagnosis, consisted of pregnant and postpartum women discharged as cured, without admission to an Intensive Care Unit (ICU) or need for invasive ventilation. These latter conditions characterize maternal severity or near-miss, according to Mantel et al. (1998) [20], and can be used as a proxy for maternal death; therefore, they were excluded from the control group.

Control data were sourced from records of pregnant and postpartum women identified in both the SIVEP-Gripe (*n* = 336) and SINASC (*n* = 254, 725) systems. Probabilistic record linkage was performed to link these databases, a method chosen due to the lack of a unique identifier across the sources. The procedure was carried out using RecLink version 3.1.8 [21].

Data processing comprised three stages: standardization, linkage, and merging. During the standardization stage, all special characters, double spaces, and diacritical marks were removed, and all letters were converted to uppercase. Two new variables, PBLOCO and UBLOCO, were created as blocking fields; these correspond to the Soundex phonetic encoding of the first and last names, respectively.

To ensure database integration, the linkage was performed according to the parameters detailed in Supplementary Table S1. Five distinct strategies were applied, varying the comparison fields (such as PBLOCO, UBLOCO, date of birth, given name, age, and municipality code). During the merging phase, pairs initially classified as false or doubtful were subjected to manual review and reclassified as matches or non-matches based on full name, date of birth, and place of residence. Pairs confirmed as false were excluded from the analysis. In the end, Strategy 1 identified 273 pairs; Strategy 2, 224; Strategy 3, 220; Strategy 4, 234; and Strategy 5, 205 pairs. After consolidation and removal of overlaps among the different strategies, a final total of 289 truly positive pairs was obtained.

After data linkage, variables common to both the case and control datasets were identified and selected, as detailed below:

(a) Sociodemographic: Marital status (with partner; without partner [including single, widowed, or separated]); years of schooling (>8; ≤8) [Corresponds to the time in years of formal education]; employment status (yes; no); area of residence (urban; rural); and Metropolitan region (yes; no).
(b) Gestational: Attendance at prenatal consultations (yes; no); number of prenatal consultations (≥6; <6); parity (nulliparous; ≥1) [Refers to the number of deliveries a woman has had, regardless of the outcome (live or stillborn), after 20 weeks of gestation]; maternal status (pregnancy; postpartum period) [Pregnancy is the period of time that begins with conception and extends until delivery. The postpartum period comprised up to 42 days after the end of pregnancy (classical maternal death) and from 43 days up to one year after the end of pregnancy (late maternal death), forming a single group in the variable “postpartum period”] [18,22].
(c) Clinical: Fever (yes; no) [The clinical threshold established by the Brazilian Ministry of Health to define fever in adults is a temperature ≥ 37.8 °C]; cough (yes; no); sore throat (yes; no); dyspnea (yes; no); respiratory distress (yes; no); oxygen saturation < 95% (yes; no); diarrhea (yes; no); vomiting (yes; no); myalgia (yes; no); heart disease (yes; no); diabetes (yes; no); obesity (yes; no).

Cases and controls were matched in a 1:3 ratio based on the year of death (2020–2021) and age group, yielding a sample of 37 cases and 112 controls. Matching by year was performed under the hypothesis that maternal healthcare conditions varied across different phases of the pandemic. Age was controlled as a known determinant of maternal mortality; this factor was controlled to mitigate potential confounding bias in the association between exposure and outcome.

Due to the fixed sample size—resulting from the inclusion of all eligible cases within the study period—the Minimum Detectable Effect (MDE) was calculated. This was performed using a specific equation for matched case–control studies [23], via Stata software (version 14.2; StataCorp LLC, College Station, TX, USA). The parameters included 37 cases matched at a 1:3 ratio, an exposure proportion among controls (P_0_) of 30%, and a correlation coefficient (*ρ*) of 0.2 between pairs [24]. With the statistical power fixed at 80% and the level of significance (α, Type I Error) at 5%, a minimum detectable Odds Ratio (*OR*) of 3.23 was estimated. This indicates that the study has 80% power to reject the null hypothesis if the true Odds Ratio in the population is equal to or greater than 3.23, given the other statistical and design parameters.

Bivariate analyses were conducted using Stata software (version 14.2; StataCorp LLC, College Station, TX, USA) to estimate *OR*s and their respective 95% confidence intervals (95% CI) through conditional logistic regression. Variables associated with the outcome at a *p*-value ≤ 0.20 were included in the Firth penalized logistic regression analysis. This technique was chosen instead of standard conditional regression to ensure the stability of the estimates in the presence of data sparsity and to avoid small-sample bias [25].

Following recommendations for unconditional models in matched study designs, the matching variables (Year and Age) were combined into a group variable and included in the model as an adjustment, ensuring correction for potential residual biases and preserving the statistical power of the sample. Variables showing an independent effect with a significance level of *p* < 0.05 were retained in the final multivariable model. The Diabetes Mellitus variable was excluded from the final adjustment due to sparsity (*n* = 7), which compromised the precision of the other variables in the model.

Associations were expressed as odds ratios (*OR*), calculated as

$$OR=e^{\beta },$$

where *β* represents the logistic regression coefficient, defined by the logit function

$$\mathrm{logit}(P)=ln\;\left(\frac{P}{1-P}\right).$$

In the bivariate matched analysis, conditional logistic regression was applied, with coefficients estimated by maximizing the conditional likelihood. Odds ratios were defined as *OR* = *e*^β^. Ninety-five percent confidence intervals (95% CI) were estimated as

exp (β±1.96×SE),

where *SE* denotes the standard error of the regression coefficient.

For multivariable analysis, Firth’s penalized logistic regression was used to reduce small-sample bias and address sparse data. The model is based on the penalized likelihood function defined as

$$L^{*}(\beta )=L(\beta )\cdot |I(\beta ){|}^{1/2}.$$

*p* values were derived using the Wald test, defined as

$$Z^{2}={\left(\frac{\hat{\beta }}{SE}\right)}^{2}.$$

In the multivariable model, statistical inference was based on Wald statistics. A two-sided significance level of 5% was adopted for all analyses.

The study was approved by the Research Ethics Committee of the Federal University of Pernambuco (Approval No. 5.246.541). Data confidentiality was strictly maintained, and the anonymity of the women, institutions, healthcare professionals, and family members who provided information during the maternal death investigations was preserved.

## 3. Results

**Figure 1:**
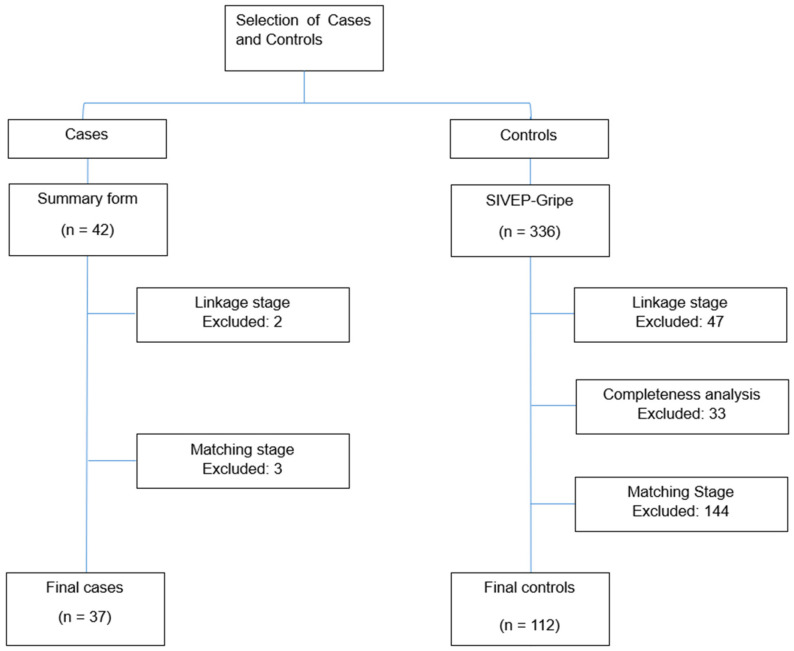
Flowchart of the selection process for pregnant and postpartum women included in the case and control groups; Pernambuco, Brazil, 2020–2021.

Of the 378 women initially identified as eligible, five were excluded from the case group: two because COVID-19 was not the underlying cause of death, and three due to the absence of age-matched controls. In the control group, 224 women were excluded: 47 because true pairs could not be established during linkage, 33 due to incomplete data, and 144 during the matching process. Consequently, the final study population consisted of 149 pregnant and postpartum women, comprising 37 cases and 112 controls (Figure 1).

Bivariate analyses indicated that the crude odds of maternal death due to COVID-19 were significantly higher among women with lower schooling (*OR*: 5.00; 95% CI: 1.10–22.69). Compared to pregnancy, significantly higher odds were observed during the postpartum period (*OR*: 39.06; 95% CI: 9.05–168.54) and among women with a parity of ≥1 (*OR*: 2.48; 95% CI: 1.07–5.76) relative to those who were nulliparous (Table 1).

Analysis of symptoms indicated that fever (*OR*: 0.07; 95% CI: 0.01–0.33) and cough (*OR*: 0.16; 95% CI: 0.06–0.43) were inversely associated with maternal death. Conversely, oxygen saturation below 95% (*OR*: 3.41; 95% CI: 1.33–8.71) was identified as a factor associated with a threefold increase in the odds of death compared to women who did not exhibit this clinical sign.

Among the pre-existing conditions evaluated, diabetes mellitus (DM) and obesity were significantly associated with maternal death in the bivariate analyses. Women with DM had 11-fold higher odds of death (*OR*: 11.00; 95% CI: 1.82–66.68), while obesity was associated with a sixfold increase in the odds of maternal death (*OR*: 6.14; 95% CI: 1.95–19.32) (Table 2).

The Firth penalized logistic regression model included the following variables: schooling, prenatal care attendance, maternal status, parity, fever, cough, oxygen saturation < 95%, and obesity.

Regression analysis identified the postpartum period (*aOR*: 80.78; 95% CI: 16.54–394.37), parity ≥ 1 (*aOR*: 5.74; 95% CI: 1.16–28.22) and oxygen saturation below 95% (*aOR*: 7.16; 95% CI: 1.37–37.44) as independent risk factors for maternal death from COVID-19. Conversely, the presence of fever was inversely associated with maternal death (*aOR*: 0.08; 95% CI: 0.01–0.42) (Table 3).

After adjustment, years of schooling, prenatal care attendance, cough, and obesity were no longer independent risk factors for the outcome. Diabetes strongly influenced the other variables and was therefore excluded from the final multivariable model to prevent interpretive bias.

## 4. Discussion

The results indicated that maternal death due to COVID-19 was associated with sociodemographic, gestational, clinical, and comorbidity-related factors. Among these, the postpartum period compared with pregnancy, parity ≥ 1, and oxygen saturation < 95% emerged as independent predictors of the outcome. The presence of fever showed an independent inverse association with the outcome, being associated with lower odds of death in this study population.

These factors were observed under differing sanitary conditions between 2020 and 2021, scenarios that were controlled in this study through matching by year of occurrence. While the first year was marked by the absence of vaccines and reliance on non-pharmaceutical interventions, such as social distancing and mask-wearing [26], the second was characterized by the introduction of mass immunization. Despite the delayed inclusion of pregnant and postpartum women in the Brazilian vaccination schedule in 2021 [13], the strategy proved effective in shifting the epidemiological profile that same year [16]. From a population perspective, vaccination is the primary mechanism for lowering the effective reproduction number (Rt), which, as demonstrated by mathematical transmission models, results in a sharp decline in infections and deaths, with this response being enhanced by the maintenance of non-pharmaceutical interventions [11,12]. Although transmission dynamics and vaccine efficacy were not direct variables in this investigation, this mitigation context explains the overall reduction in maternal mortality from 2021 onwards. By isolating this temporal effect through matching, the study demonstrates that, regardless of the general sanitary landscape, individual factors remained determinants of maternal death.

Initially, the analysis indicated an association between maternal death from COVID-19 and lower educational attainment. However, after adjustment for other factors, this association was no longer statistically significant. In contrast, a study on maternal mortality from COVID-19 conducted in Latin America [10] as well as another secondary data study in Brazil [27] reported a higher frequency of deaths among women with lower educational levels. Low educational attainment is often linked to adverse socioeconomic conditions, limited access to health information, and reduced access to healthcare services [28], vulnerabilities elements that were intensified during the pandemic.

Evidence from Italy shows that socioeconomic deprivation modulated both the spread of COVID-19 and the impact of restrictive measures, limiting the ability of vulnerable populations to adhere to social distancing and to maintain adequate access to healthcare services [29]. Moreover, the economic instability and logistical barriers imposed during the public health emergency altered patterns of seeking reproductive healthcare, deepening pre-existing inequalities [30]. In this context, poverty—analyzed across multiple dimensions—remains a structural determinant of maternal mortality [31], even though its effect may be partially mediated by clinical and healthcare-related factors.

Regarding maternal status, the postpartum period was identified as a significant independent risk factor for maternal death relative to pregnancy, a finding consistent with previous literature [14,32,33]. A Brazilian study on COVID-19-related severe acute respiratory syndrome (SARS) reported similar results when comparing pregnant and postpartum women, identifying that those in the puerperium had a higher likelihood of ICU admission, invasive respiratory support, and death [33]. This increased risk represents a complex interplay of clinical and organizational challenges. From a clinical perspective, the puerperium is a period of intense physiological transition, in which the inherent prothrombotic state is exacerbated by the inflammatory response to COVID-19, increasing the risk of thromboembolism [34]. Furthermore, the high rate of cesarean deliveries in Brazil—which further increased during the pandemic—imposes surgical trauma and metabolic stress that potentially worsen the prognosis of SARS-CoV-2 infection [5,35]. Organizationally, the increased likelihood of death in the postpartum period may reflect failures in the continuum of care, manifested by difficulties in accessing health services, disorganization of the obstetric care network, and delays in specialized care. In the context of the COVID-19 public health emergency in Brazil, structural barriers and shortages of human and material resources further intensified delays in obstetric care, contributing to the worsening of maternal outcomes [36,37,38]. These findings highlight the importance of close monitoring and targeted care for women with COVID-19 during the puerperium in this region of Brazil.

The magnitude of the association observed for the postpartum period, although clinically plausible, should be interpreted with caution. The wide confidence interval reflects the limitations imposed by the sample size and the distribution of cases and controls during the study period, characterizing a scenario of sparse data. Nevertheless, the persistence of strong statistical significance (*p* < 0.001) and consistency with previous studies support the validity of this finding, highlighting the need for intensive monitoring of women in the postpartum period.

Another gestational factor independently associated with the analyzed outcome was increased parity. There is a limited number of studies that have investigated the impact of this variable on maternal mortality due to COVID-19 [39,40]. However, existing evidence indicates a predisposition to adverse outcomes; previous research [41,42], identified a link between multiparity and disease severity. It was demonstrated in a UK national cohort that a significant proportion of pregnant women hospitalized with COVID-19 were multiparous [39]. The underlying mechanisms are not fully elucidated, but one hypothesis is that multiparity is more common among older women, who may already present with comorbidities [42]. Maternal mortality is deeply influenced by the cumulative effect of obstetric history and social determinants on women’s health [40]. However, an international study analyzing data from 926 pregnant women with COVID-19 demonstrated that parity was not significantly associated with case severity [43]. In our study, the association of the variable with the presence of comorbidities and advanced age was not analyzed.

Among clinical signs and symptoms, oxygen saturation below 95% was identified as an independent risk factor for maternal death from COVID-19, consistent with previous studies [27,38,44]. Beyond the obstetric context, low oxygen saturation is recognized as a universal hallmark of COVID-19 severity, directly reflecting the extent of pulmonary involvement and the systemic inflammatory response. In Latin America, a multicenter study of 447 maternal deaths identified the most frequent respiratory dysfunctions, highlighting hypoxemia (SpO_2_ ≤ 90%) and severe tachypnea [10]. The increased vulnerability of obstetric patients is attributed to physiological and anatomical changes that compromise pulmonary function and reduce tolerance to hypoxia [45]. When combined with SARS-CoV-2 infection, these factors may act synergistically, increasing the risk of severe complications. A decline in oxygen saturation may, therefore, serve as an early warning sign of clinical deterioration in pregnant and postpartum women [46].

Fever was observed in half of the cases and was associated with reduced odds of maternal death. However, this finding should be interpreted with caution, especially in the context of an observational study based on secondary data. The possibility of underreporting, particularly in severe cases or sudden deaths where clinical records may be incomplete, cannot be ruled out. In addition, the presence of fever may have acted as an early warning sign, prompting immediate care-seeking and influencing the outcome. Therefore, this inverse association may reflect healthcare-seeking dynamics and data recording patterns rather than a true biological protective effect.

Despite these caveats, our finding is consistent with a cohort study by Costa et al. which identified fever at hospital admission as a protective factor (*aOR*: 0.63; CI 95%: 0.42–0.96) for death among 996 COVID-19 patients with a history of vaccination [47]. Similarly, a meta-analysis of COVID-19 outcomes reported a lower prevalence of fever in critically ill patients and those with fatal outcomes (*OR*: 0.56; 95% CI: 0.38–0.82) [48]. These findings are supported by evidence that fever enhances immune efficacy during infections by stimulating both innate and adaptive immune responses and reducing viral replication [49,50]. Nevertheless, other studies have reported associations between fever and adverse outcomes in both the general [51] and obstetric populations [52].

In the analysis of comorbidities, obesity was not identified as an independent risk factor for the outcome. This finding aligns with other studies [38,43], but contrasts with reports in the literature that highlight obesity as a risk factor [14,32]. A Brazilian study of 978 women with COVID-19 reported a 2.3-fold higher risk of maternal death among those with obesity [14]. The complications associated with obesity are often linked to metabolic syndrome, which can lead to hypertension and diabetes, resulting in macro- and microvascular damage and endothelial dysfunction [53], thereby exacerbating COVID-19 severity [54]. In our study, the low prevalence of diabetes and cardiovascular disease in the sample may explain why obesity alone was not associated with maternal death.

Diabetes showed an association with the outcome in the bivariate analysis. However, it was excluded from the final multivariable model due to numerical instability caused by data sparsity (*n* = 7), which compromised the precision of the other estimates. The exclusion of a clinically relevant comorbidity may introduce residual confounding, which represents an inherent limitation of studies of rare events in restricted population samples. The results differ from others in the literature that found an association with severe and fatal outcomes in pregnant women [43] and the general population with COVID-19 [48]. The reduced sample size of pregnant women with the disease in this study may explain this divergence.

Our findings also differ from population-based studies that described diabetes as an associated risk factor. The association between cardiovascular and metabolic diseases and a higher risk of complications and death from COVID-19 is widely recognized and well-substantiated by physiopathological mechanisms. However, the presence of only one of these comorbidities may not be sufficient to classify a patient as high risk [55]. According to the Charlson comorbidity index, the severity of the comorbidity is more relevant than its mere presence [56].

This study has limitations inherent to the use of secondary databases and to the analysis of epidemiologically rare events in restricted populations. The absence or incompleteness of variables in official records—such as laboratory test results, specific obstetric outcomes, and comorbidities—combined with the exclusion of inconsistent records, restricted the analysis to the information available in the information systems. Additionally, data sparsity for the variable Diabetes Mellitus (*n* = 7) required its exclusion to ensure numerical stability and convergence in the Firth regression. Although necessary, this omission may have introduced residual confounding, potentially overestimating the odds ratios of the remaining variables. The wide confidence interval observed for the ‘postpartum period’ reflects limited precision due to the sample size, warranting caution in interpreting the magnitude of this effect. Furthermore, the risk of underreporting signs and symptoms due to manual data entry may mask the impact of underlying factors. Finally, given the geographic scope and the specificity of the sample, the extrapolability of these findings to other contexts should be undertaken with caution.

## 5. Conclusions

This study presents an analysis of the factors associated with maternal death during the COVID-19 pandemic in Pernambuco, Brazil. The findings reinforce that maternal death is a multifactorial phenomenon. Based on a sample of 149 pregnant and postpartum women (37 deaths and 112 survivors), our findings reveal that maternal death was associated with the postpartum period (*aOR*: 80.78), multiparity (*aOR*: 5.74), and oxygen saturation below 95% (*aOR*: 7.16). In contrast, the presence of fever showed an inverse association with the outcome (*aOR*: 0.08), suggesting either underreporting of the symptom or a protective effect that warrants investigation in future studies.

The relevance of this investigation lies in identifying clinical and obstetric vulnerability profiles in a region heavily impacted by the health crisis. The robustness of the evidence is sustained using probabilistic linkage between official databases; the use of maternal death data following investigation and discussion by a technical committee—ensuring greater information accuracy—and the application of Firth’s penalized logistic regression, which provided statistical stability to the estimates despite the rarity of the analyzed events.

In summary, the knowledge acquired from past crises contributes to the improvement of public health strategies and clinical management protocols, aiming to mitigate preventable maternal deaths in future public health emergencies.

## Figures and Tables

**Table 1:** Bivariate analyses of sociodemographic and gestational factors associated with maternal death from COVID-19; Pernambuco, Brazil, 2020–2021.

Exposure	Maternal Death		*OR* (95% CI)	*p*-Value
	Yes *n* (%) (*n* = 37)	No *n* (%) (*n* = 112)		
Marital status				
With a partner	20 (55.0%)	52 (46.4%)	1.00	
Without a partner	17 (45.0%)	60 (53.6%)	0.91 (0.36–2.25)	0.841
Years of Schooling				
>8 years	3 (8.1%)	18 (16.0%)	1.00	
≤8 years	34 (91.9%)	94 (84.0%)	5.00 (1.10–22.69)	0.037
Employment status				
Yes	25 (67.6%)	55 (49.0%)	1.00	
No	12 (32.4%)	57 (51.0%)	0.66 (0.28–1.51)	0.330
Area of residence				
Urban	32 (86.5%)	102 (91.0%)	1.00	
Rural	5 (13.5%)	10 (9.0%)	1.32 (0.38–4.50)	0.660
Metropolitan Region				
Yes	19 (51.4%)	64 (57.0%)	1.00	
No	18 (48.6%)	48 (43.0%)	1.03 (0.45–2.35)	0.929
Prenatal consultations				
Yes	34 (92.0%)	109 (97.0%)	1.00	
No	3 (8.0%)	3 (3.0%)	4.22 (0.62–28.42)	0.138
Number of prenatal consultations				
≥6	20 (54.0%)	61 (54.5%)	1.00	
<6	17 (46.0%)	51 (45.5%)	0.87 (0.41–1.87)	0.735
Maternal status				
Pregnancy	4 (11.0%)	103 (92.0%)	1.00	
Postpartum period	33 (89.0%)	9 (8.0%)	39.06 (9.05–168.54)	<0.001
Parity				
Nulliparous	8 (22.0%)	51 (45.5%)	1.00	
≥1	29 (78.0%)	61 (54.5%)	2.48 (1.07–5.76)	0.034

**Table 2:** Bivariate analyses of signs, symptoms, and comorbidities associated with maternal death from COVID-19; Pernambuco, Brazil, 2020–2021.

Exposure	Maternal Death		*OR* (95% CI)	*p*-Value
	Yes *n* (%) (*n* = 37)	No *n* (%) (*n* = 112)		
Fever	10 (27.0%)	67 (59.8%)	0.07 (0.01–0.33)	<0.001
Cough	19 (51.4%)	87 (77.6%)	0.16 (0.06–0.43)	<0.001
Sore throat	3 (8.1%)	25 (22.3%)	0.29 (0.07–1.07)	0.064
Dyspnea	21 (56.8%)	55 (49.1%)	0.75 (0.30–1.87)	0.547
Respiratory distress	17 (46.0%)	37 (33.0%)	1.14 (0.50–2.63)	0.742
Saturation < 95%	19 (51.4%)	21 (18.7%)	3.41 (1.33–8.71)	0.010
Diarrhea	1 (2.7%)	6 (5.3%)	1.12 (0.13–9.70)	0.915
Vomiting	2 (5.4%)	12 (10.7%)	0.62 (0.12–3.09)	0.566
Myalgia	5 (13.5%)	7 (6.3%)	2.21 (0.57–8.48)	0.248
Heart disease	3 (8.1%)	3 (2.7%)	2.90 (0.53–15.61)	0.215
Diabetes	4 (10.8%)	3 (2.7%)	11.02 (1.82–66.68)	0.009
Obesity	11 (29.7%)	6 (5.4%)	6.14 (1.95–19.32)	0.002

**Table 3:** Firth penalized multivariable logistic regression analysis of gestational factors, signs, and symptoms associated with maternal death from COVID-19; Pernambuco, Brazil, 2020–2021.

Exposure	Unadjusted			Adjusted		
	*OR*	95% CI	*p*-Value	*aOR*	95% CI	*p*-Value
Maternal status						
Pregnancy	1.00					
Postpartum period	39.06	9.05–168.54	<0.001	80.78	16.54–394.37	<0.001
Parity						
Nulliparous	1.00					
≥1	2.48	1.07–5.76	0.034	5.74	1.16–28.22	0.031
Oxygen Saturation < 95%	3.41	1.33–8.71	0.010	7.16	1.37–37.44	0.020
Fever	0.07	0.01–0.33	0.001	0.08	0.01–0.42	0.002

## Data Availability

Restrictions apply to the availability of these data. The data were obtained under a confidentiality agreement with Health Institutions and can only be made available upon permission from the involved units.

## Supplementary Materials

The following supporting information can be downloaded at: https://www.mdpi.com/article/10.3390/diseases14020071/s1, Table S1: Strategies, parameters, and results of the probabilistic linkage between the SIVEP-Gripe and SINASC databases for the formation of the control group, Pernambuco, Brazil, 2020–2021.
