# Supplementary material for: Factors Associated with Maternal Mortality from COVID-19 in Pernambuco, Brazil (2020–2021): A Case–Control Study
Source: Diseases. 2026 Feb 14;14(2):71. doi: 10.3390/diseases14020071 (PMC12939328; doi:10.3390/diseases14020071)
Supplement: Supplementary file 1 [file diseases-14-00071-s001.zip › diseases-4051483-supplementary.pdf]

Supplementary table S1. Strategies, parameters, and results of the probabilistic linkage between the SIVEP-Gripe and SINASC databases for the formation of the control group, Pernambuco, Brazil, 2020–2021.

Strategy*	Sensibilidade (m_i)	1 – Especificidade (u_i)	Threshold	Range	Score	Related Files		Total Pairs**	True Positives***
**Strategy 1**						**Sivep-gripe**	**Sinasc**		
Pbloco	95%	5%	85%						
Ubloco	95%	5%	85%	-11.66 to 11.66	11.66	336 records ****	254,725 records	295	273
Date of birth	90%	10%	65%						
**Strategy 2**						**Sivep-gripe**	**Sinasc**		
Given name	95%	5%	85%						
Pbloco	95%	5%	85%	-15.91 to 15.91	15.91	336 records ****	254,725 records	281	224
Ubloco	95%	5%	85%						
Age	90%	10%	65%						
**Strategy 3**						**Sivep-gripe**	**Sinasc**		
Given name	95%	5%	85%						
Date of birth	90%	10%	65%	-11.66 to 11.66	11.66	336 records ****	254,725 records	220	220
Municipality code	95%	5%	85%						
**Strategy 4**						**Sivep-gripe**	**Sinasc**		
Given name	95%	5%	85%						
Pbloco	95%	5%	85%	-15.91 to 15.91	15.91	336 records ****	254,725 records	234	234
Ubloco	95%	5%	85%						
Date of birth	90%	10%	65%						
**Strategy 5**						**Sivep-gripe**	**Sinasc**		
Given name	95%	5%	85%	10.58 to 10.58					
Date of birth	90%	10%	65%		10.58	336 records ****	254,725 records	205	205
Age	90%	10%	65%						
**Total pairs*****									**289**

Strategy 1*: *soundex* of the first name; *soundex* of the last name; date of birth. Strategy 2 Given name; *soundex* of the first name; *soundex* of the last name. Strategy 3 Given name; date of birth; municipality code. Strategy 4 Given name; *soundex* of the first name; *soundex* of the last name; date of birth. Strategy 5 Given name; date of birth; age. ** Total number of pairs identified through probabilistic linkage between the SIVEP-Gripe and SINASC databases. ***Total number of pairs, after excluding uncertain matches and non-pairs (records with discrepancies in name, date of birth, and/or municipality). **** Pregnant and postpartum women with a positive PCR test for COVID-19, who were not admitted to the ICU, did not require invasive ventilatory support, and were discharged as cured (2020–2021). ***** Total number of true positive pairs after consolidation and removal of overlaps among the different strategies.
